# Diagnosing Pneumonia in the Obese: Are Plain Radiographs Enough?

**DOI:** 10.1017/ash.2024.306

**Published:** 2024-09-16

**Authors:** Alwyn Rapose

**Affiliations:** Reliant Medical Group and Saint Vincent Hospital

## Abstract

**Introduction:** The obesity pandemic presents numerous challenges in radiography, including patients exceeding the weight limits of imaging equipment, difficulties in palpating anatomical landmarks and inadequate penetration resulting in inaccurate diagnoses and need for repeat imaging resulting in increased radiation exposure. Pneumonia is a common reason for hospitalization, but identifying an infiltrate on the chest radiograph (CXR) in obese patients can be challenging. Conversely, motion artefacts may be misinterpreted as areas of pneumonia resulting in over-diagnosis and consequently the overuse of antibiotics. This study evaluated the diagnostic utility of CXR for identifying pneumonia in obese patients by comparing it with computed tomography (CT scan) performed within 72 hours. We also evaluated the impact on initiation or discontinuation of antibiotics – a very important aspect of antibiotic stewardship. **Methods:** All patients admitted between July 2020 and 2022 with a diagnosis of pneumonia who underwent both CXR and chest CT scan within a 72-hour window were included. Patients were divided into two groups obese and non-obese based on BMI. The results were evaluated to determine the concordance between the two modalities in diagnosing pneumonia, as well as its influence on antibiotic therapy. **Results:** A total of 320 patients were included, with a mean age of 65.3 years. 146 (45.6%) were male, and 174 (54.4%) were female. 202 (63.1%) were classified as obese (BMI > 30). CXR was performed as first modality in 313 (97.8%) cases, while 7 (2.2%) underwent CT scan first. In the obese group the overall concordance between the 2 modalities for diagnosing pneumonia was 67.5%. In the non-obese group the concordance was 80.2% (p < 0 .001). Among the obese patients who underwent CXR first, 11 (5%) had antibiotics discontinued after the CT scan results, while the number was 4 (3%) in the non-obese group. Additionally, 3 patients in the obese group had antibiotics initiated after the CT scan. **Conclusions:** Obesity poses unique challenges to healthcare facilities and imaging equipment. Diagnosing pneumonia in obese patients using CXR alone may result in over-diagnosis. This may lead to unnecessary antibiotic use and delayed diagnosis of alternate disease, or in some cases, missing a pneumonia and under-treatment. A chest CT scan is more sensitive and may be more helpful to identify a pneumonia accurately in these patients and thus facilitate appropriate antibiotic use.

